# Effect of implementing an anesthesia nurse checklist in a safety and teamwork climate: quasi-experimental study^*^

**DOI:** 10.1590/1980-220X-REEUSP-2021-0471en

**Published:** 2022-09-19

**Authors:** Cassiane de Santana Lemos, Vanessa de Brito Poveda

**Affiliations:** 1 Universidade de São Paulo, Escola de Enfermagem, Departamento de Enfermagem Médico-Cirúrgica, São Paulo, SP, Brazil.

**Keywords:** Perioperative Nursing, Anesthesia, General, Patient Safety, Cecklist, Quality of Health Care, Patient Care Team, Enfermería Perioperatoria, Anestesia General, Seguridad del Paciente, Lista de Verificación, Calidad de la Atención de Salud, Grupo de Atención al Paciente, Enfermagem Perioperatória, Anestesia Geral, Segurança do Paciente, Lista de Checagem, Qualidade da Assistência à Saúde; Equipe de, Assistência ao Paciente

## Abstract

**Objective:**

To evaluate the effect of implementing a Patient safety checklist: nursing in anesthetic procedure on the perception of safety climate and team climate of nurses and anesthesiologists from an operating room.

**Method:**

Quasi-experimental study held in the operating room of a hospital in Brazil with a sample of nurses and anesthesiologists. The outcome was evaluated through the instruments “Safety Attitudes Questionnaire/Operating Room Version” and “Team Climate Inventory”, applied before and after the implementation of a Patient safety checklist: nursing in anesthetic procedure by nurses. The mixed effects linear regression model was used to analyse the effect of the implementation.

**Results:**

Altogether, 19 (30.2%) nurses and 44 (69.8%) anesthesiologists participated in the study, implementing the Patient safety checklist: nursing in anesthetic procedure in 282 anesthesias. The Safety Attitudes Questionnaire/Operating Room Version score changed from 62.5 to 69.2, with modification among anesthesiologists in the domain “Perception of management” (p = 0.02). Between both professionals, the Team Climate Inventory score increased after the intervention (p = 0.01).

**Conclusion:**

The implementation of the Patient safety checklist: nursing in anesthetic procedure changed the perception score of safety and teamwork climate, improving communication and collaborative work.

## RESUMEN

**Objetivo:**

Evaluar el efecto de la implementación de un *checklist* de seguridad del paciente: enfermería en el procedimiento anestésico (PSC/NAP) sobre la percepción del clima de seguridad y de equipo de enfermeros y anestesiólogos de un centro quirúrgico.

**Método:**

Estudio casi experimental realizado en el centro quirúrgico de un hospital de Brasil con una muestra de enfermeros y anestesiólogos. El resultado fue evaluado por medio de los instrumentos “Safety Attitudes Questionnaire/Operating Room Version (SAQ/OR)” y “Team Climate Inventory (TCI)”, aplicados antes y después de la implementación de un PSC/NAP por los enfermeros. El modelo de regresión lineal de efectos mixtos fue utilizado para analizar el efecto de la implementación.

**Resultados:**

En total, 19 (el 30,2%) enfermeros y 44 (el 69,8%) anestesiólogos participaron del estudio, implementando el PSC/NAP para 282 anestesias. El escore del SAQ/OR cambió de 62,5 a 69,2, con modificación entre los anestesiólogos en el dominio “Percepción del manejo” (p = 0,02). Entre los dos profesionales, el escore del TCI aumentó después de la intervención (p = 0,01).

**Conclusión:**

La implementación del PSC/NAP alteró el escore de percepción de seguridad y clima de trabajo en equipo, mejorando la comunicación y el trabajo colaborativo.

## RESUMO

**Objetivo:**

Avaliar o efeito da implementação de um checklist de segurança do paciente: enfermagem no procedimento anestésico sobre a percepção do clima de segurança e clima de equipe de enfermeiros e anestesiologistas de um centro cirúrgico.

**Método:**

Estudo quase experimental realizado no centro cirúrgico de um hospital do Brasil com uma amostra de enfermeiros e anestesiologistas. O desfecho foi avaliado por meio dos instrumentos *“Safety Attitudes Questionnaire/Operating Room Version” e “Team Climate Inventory*”, aplicados antes e após a implementação de um checklist de segurança do paciente: enfermagem no procedimento anestésico pelos enfermeiros. O modelo de regressão linear de efeitos mistos foi utilizado para analisar o efeito da implementação.

**Resultados:**

Ao todo, 19 (30,2%) enfermeiros e 44 (69,8%) anestesiologistas participaram do estudo, implementando o checklist de segurança do paciente: enfermagem no procedimento anestésico em 282 anestesias. O escore do *Safety Attitudes Questionnaire/Operating Room* mudou de 62,5 para 69,2, com modificação entre os anestesiologistas no domínio “Percepção da gestão” (p = 0,02). Entre os dois profissionais, o escore do *Team Climate Inventory* aumentou após a intervenção (p = 0,01).

**Conclusão:**

A implementação do *Patient safety checklist: nursing in anesthetic procedure* modificou o escore de percepção de clima de segurança e clima de trabalho em equipe, melhorando a comunicação e o trabalho colaborativo.

## INTRODUCTION

Initiatives for patient safety in anesthesia contributed over the years to the global reduction of mortality risk associated with anaesthetic-surgical procedures, which improved the quality of perioperative care. These initiatives involved advanced training, certification, and teamwork professionals’ education, improving techniques, medications, patient monitoring, risk assessment standards for surgery, development and application of care protocols^(1,2)^.

However, adverse events and incidents associated with the anesthetic procedure are still present in the reality of perioperative care, related to human errors, communication, and team­work failure^(3)^. Evidence showed that in 747 cases reviewed, 196 (26.2%) events were related to human causes^(4)^, and another study evidenced that among 511 anesthetic procedures, 111 (21.7%) adverse events happened, of which 53 (31%) occurred because of human factors, errors in drug administration, and equipment failures^(5)^.

Therefore, organizational actions such as administrative decisions, institutional safety culture, and managerial processes of health institutions can influence care safety and teamwork. Thus, the institutional safety culture encompasses teamwork, communication, and leadership, including the institution’s values and processes, directly related to the safety climate^(6)^.

Safety climate is defined as the workers’ perception of safety at the place of performance^(6)^. Otherwise, the teamwork climate is characterized as the perceptions shared by workers about innovation, defined as the intentional introduction and application of new ideas, processes and/or products in the team or organizational institution, which are relevant and beneficial to performance of the group, organization or society^(7)^.

Health care involves different levels of health systems. The dimensions are related to efficiency with which the workers develop their activities, patient care effectiveness, equity, and opportunity of access to the services for the society, which can impact quality and safety of care^(8)^. Also, multi-professional discussions about adverse events and the development of care protocols can influence interventions quality and safety^(2)^.

In 2008, the World Health Organization (WHO) proposed the implementation of the safe surgery checklist, which generated changes in the perioperative quality of care in hospital institutions, directly connected with the reduction of complications^(9,10)^. The improved communication among professionals generated an enhanced perception of a safety climate related to care and teamwork^(11,12,13)^, due to an increased sharing of case critical information, resulting in better decision-making, team coordination, openness about knowledge gaps, and team cohesion^(9)^.

The implementation of patient safety checklists revealed an increased perception of the safety climate among the workers^(9,13–14)^, which indicates a positive recognition that the actions and measures introduced in their practice promote safer care to the patient.

The safe surgery checklist reduces postoperative infections, cardiac complications, bleeding, and leads to major adherence to operation room safety procedures, such as prophylactic antibiotics and installation of a thermal blanket^(15)^.

The use of checklists in anesthesia, whether in routine or emergency situations, seems to improve anesthetic processes and decrease perioperative morbidity and mortality^(15)^. Furthermore, anesthesia checklists improve information exchange, communication, and professional performance^(16)^.

In Brazil, there is a lack of standardization of anesthetic nursing care^(17,18)^, which could jeopardize health assistance quality and compromise the perception of the safety and teamwork climate among the workers involved. In this context, the development of checklists or guidelines for anesthetic nursing care can enhance patient safety by preventing adverse events due to the standardization of nursing team daily routines and strengthened record of actions. The Patient safety checklist: nursing in anaesthetic procedure (PSC/NAP)^(19)^ is a Brazilian validated tool developed to help nurses during nurse assistance to general anesthesia, consisting of nursing care items to be carried out by nurses in the pre-induction, induction, and reversion of general anesthesia.

Thus, the objective of this study was to evaluate the effect of the implementation of a Patient safety checklist: nursing in anesthetic procedure (PSC/NAP) on the perception of safety climate and team climate of nurses and anesthesiologists from an operating room. We hypothesized that implementing a nursing care protocol during the anesthetic procedure can change the workers’ perception about the safety and the teamwork climate in the operating room.

## METHOD

### Study Design

The study had a quasi-experimental, quantitative approach with pre-test/post-test design. We considered the “Safety Attitudes Questionnaire/Operating Room Version (SAQ/OR)” and “Team Climate Inventory (TCI)” pre- and post-test nurses and anesthesiologists’ scores as dependent variables, and the implementation of a nursing checklist in anesthesia (PSC/NAP) (intervention) an independent variable.

Nurses and anesthesiologists were evaluated by SAQ/OR and TCI before and after the implementation of a PSC/NAP (intervention) by the nurses.

The PSC/NAP was applied for six months by assistant nurses, in a convenience sample of 281 patients older than 18 years and who underwent surgical procedures under general anesthesia.

The six months application of the PSC/NAP was defined based on the recommendation that evidence implementation projects must perform an audit of the implementation process after six months to achieve 50 to 80% compliance to the new standards of practice^(20)^.

This manuscript adheres to the TREND guideline.

### Population Accessible

Nurses and anesthesiologists working in the operating room. The nursing team consisted of 29 nurses, of whom 20 performed direct assistance in the operating room, and 45 anesthesiologists.

### Selection Criteria

The inclusion criteria were being a nurse at the operating room with a minimum of one year in the sector and implementation of direct assistance to the patient in the surgery room; being an anesthesiologist of the anesthesia service provider and having a minimum of one-year activity in the sector. Exclusion criterium was nurses who had not participated in the guidance for the use of the checklist.

### Sample

Convenience sampling was composed of 19 nurses and 44 anesthesiologists, who were invited to collaborate with the study. One nurse refused to participate, and one anesthesiologist was on vacation at the beginning of the data collection.

Due to the interprofessional and collaborative work between nurses and anesthesiologists in the operating room, the anesthesiologists were included in the sample to evaluate if the exposition of the effect of PSC/NAP on anesthesia nursing work affects the perception of safety and team climate.

### Data Collection

**Figure 1:**
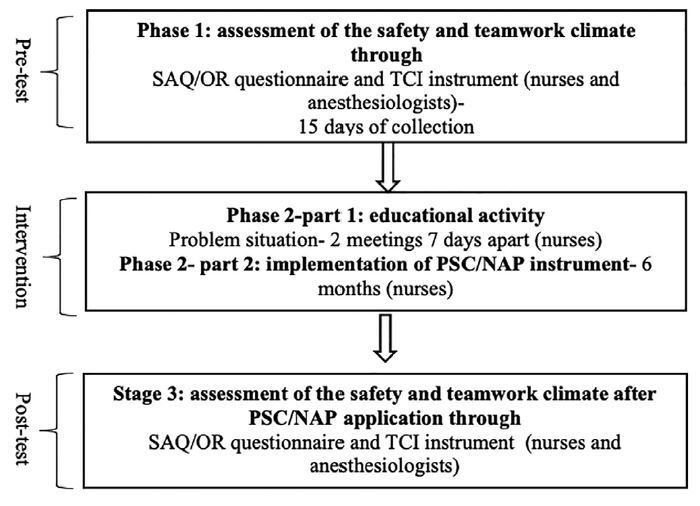
Study phases

The study was conducted in a private hospital of the municipality of São Paulo, Brazil, from December 2017 to July 2018. The data collection site was defined due to the ratio of nursing workforce/operating room and adherence to protocols of quality of assistance in the operating room. Figure 1 shows all phases of data collection.

Before implementing the PSC/NAP, nurses participated in an educational activity addressing nursing in anesthesia based on problem-based learning. Two meetings of one hour each were held in a training room, where data collection took place. According to their work shift, the nurses were divided into three groups: two with six nurses and one with seven nurses^(21)^. The instrument defined as PSC/NAP^(19)^ was presented to the participant nurses by the researcher, with guidance on its application at the end of the second meeting.

Additional information can be documented, such as the worker and the patient identification, patient’s physical status classification (ASA), type of anesthesia and surgery, start and end time of the anesthetic-surgical procedure, and motives for not performing some recommended care.

PSC/NAP was applied during six months, in all work shifts (morning, afternoon, night) by 19 nurses during general anesthesia procedures performed by an anesthesiologist of the anesthesia service provider.

The PSC/NAP is a tool consisting of nursing care items to be carried out by nurses in the three periods of anesthesia (pre-induction, induction, and reversion of anesthesia). The PSC/NAP was validated in a previous study, obtaining a content validity index (CVI), ranging from 80% to 100% between the evaluated items^(19)^.

The Instruments SAQ/OR and TCI were applied before and after the PSC/NAP implementation to the nurses and anesthesiologists participating in the study.

To assess the safety climate in the team, the workers answered a SAQ/OR^(14)^, validated and adapted cross-culturally to the Portuguese language spoken in Brazil. The questionnaire was chosen because it was validated in a previous study^(22)^, being a suitable tool to measure the safety climate in the environment of the surgical centre.

The Portuguese validated version of SAQ/OR questionnaire is composed of 40 items, distributed in six domains (safety climate, perception of management, stress recognition, working conditions, communication in the environment of the operating room, and perception of the worker’s performance), and six factors (medical errors approach, job satisfaction, reporting errors, personal problems of the staff and miscommunication, hospital administration, and the surgeon’s coordination of the operating room)^(14)^. This study used the version SAQ/OR questionnaire, specific to operating room, which does not include teamwork climate as domain, different from the generic SAQ version^(14)^.

The domains of the SAQ/OR questionnaire are presented in the form of questions and statements by means of a Likert scale with scores of 0 to 100 points, represented by: totally disagree (0 points); partially disagree (25 points); neutral (50 points); partially agree (75 points); and totally agree (100 points). Values higher than or equal to 75 are considered a positive perception of patient safety^(14)^.

To evaluate the teamwork climate, the workers answered the instrument TCI validated for the Portuguese language spoken in Brazil^(23)^. The instrument was validated in a previous study^(7)^, being a suitable tool to assess the teamwork climate.

The TCI consists of 38 items and assesses the teamwork climate by four dimensions: participation in the team, support for new ideas, team goals, and task orientation. To the domains “participation in the team” and “support for new ideas”, the factors are composed of questions and statements presented on a Likert scale, with five answer alternatives: (5) strongly agree, (4) agree, (3) neither agree/neither disagree, (2) disagree and (1) strongly disagree. The domain “team goals” and “task orientation” have seven alternatives that indicate the agreement with the options described, measured respectively as (6–7) completely, (3–5) somewhat, or (1–2) no way, and (6–7) a lot, (3–5) to some extent or (1–2) a little^(23)^. Considering all domains, the score of the TCI scale varies from 38 to 226 points, whereby the higher the score, the better perception of teamwork climate. The Brazilian validated version of TCI did not establish cut points.

### Statistical Analysis

The software R, version 3.5.1. was used in data analysis of the present study, setting a level of significance of 5%.

Categorical variables were presented as absolute and relative frequencies, and the numerical variables were presented in the form of measures of central tendency (mean and standard deviation-SD). The total score and scores of each domain of the questionnaire SAQ/OR and the instrument TCI were evaluated according to the position and pre- and post-intervention exposure periods of the PSC/NAP.

The mean differences in the instrument domains were analysed using a Linear Mixed-Effects Regression Model (LMM), taking into account the exposure period and the position. The interaction period and position were defined to measure the difference pre- and post-intervention regarding safety and teamwork perception between nurses and anesthesiologists. The main effect of position evaluated the constant difference between positions (nurses and anesthesiologists), regardless of the period. The main effect of the exposure period measured the difference before and after the intervention, regardless of the worker’s position.

To assess the effect of the implementation of the PSC/NAP on the SAQ/OR questionnaire and the TCI instrument, the LMM was used, fixing factors of interest: post-intervention total score, items executed from the PSC/NAP, worker’s age, nurse’s position, professional experience, male sex, number of PSC/NAP completed by the nurse and exposure of the anesthesiologist to the PSC/NAP, all factors interacting with the period (pre- and post-intervention).

### Ethical Aspects

The study was approved in October 2017 by the Ethics and Research Committee of the USP School of Nursing, under number 2.340.000, in accordance with Resolution 466/12. All participants provided their Signed Informed Consent Forms before the study.

## RESULTS

A total of 63 workers were included in the study, 19 (30.2%) nurses and 44 (69.8%) anesthesiologists. More than half the anesthesiologists were men (37; 84.09%) and had mean age of 43.86 (SD = 12.22) years. On the other hand, 17 (89.47%) nurses were women and had mean age of 33.26 (SD = 3.78) years, with partial shift (11; 57.89%). Nurses had a mean of 5.07 (SD = 4.06) years of hospital experience, while anesthesiologists had a mean of 14.54 (SD = 11.56) years.

Nurses applied the PSC/NAP in 281 anaesthetic procedures, 148 (52.48%) submitted to balanced general anesthesia, performed on 171 (60.64%) ASA II patients. The surgical procedure had a mean duration of 2.69 hours ±2.24, and the anaesthetic procedure had a mean of 1.32 hours ±1.02, with 33.80% of the surgical procedures being a general surgery.

Table 1 shows the comparison of the pre- and post-intervention score of the workers’ perceptions, according to the domains of the questionnaire SAQ/OR.

**Table 1.** Comparison of preoperative and postoperative score of the workers’ perceptions, by position, according to the domains of the Safety Attitudes Questionnaire/Operating Room Version (SAQ/OR) questionnaire – São Paulo, SP, Brazil, 2018.

Domains	Position	Period		p-values		
		Pre mean (SD*)	Post mean (SD)	Exposure period	Position	Interaction: period and position
Total SAQ	N	62.5 (14.1)	64.7 (12.5)	0.08	0.18	0.93
	A	66.6 (10.9)	69.2 (10.7)			
Safety climate	N	77 (13.7)	80.3 (11.5)	0.36	0.97	0.69
	A	78.3 (13.8)	80.2 (15.6)			
Perception of management	N	62.8 (16.7)	62.9 (16.9)	0.0002	0.0007	0.02
	A	68.5 (15.7)	79.3 (16.2)			
Stress recognition	N	53.9 (31.3)	56.6 (29.2)	0.49	0.33	0.93
	A	61.6 (26.1)	64.3 (24.7)			
Working conditions	N	64.5 (20.4)	73.7 (16.8)	0.36	0.82	0.08
	A	72.6 (15.5)	74.6 (16.2)			
Communication environment	N	80.3 (20.1)	85.1 (10.2)	0.23	0.14	0.55
	A	87.5 (9.81)	90.1 (9.68)			
Perception of professional performance	N	29.9 (28.2)	32.5 (27.1)	0.20	0.007	0.23
	A	18.8 (21.3)	15.4 (18.8)			

* SD = standard deviation; N: nurse; A: anesthesiologist.

There was a slight variation in the total score of the SAQ/OR questionnaire between the pre- and post-intervention, and the mean was higher among anesthesiologists. The average score of the two professional categories was between 62.5 and 69.2 (Table 1).

The assessment of the interaction of the intervention exposure period (PSC/NAP) and the position indicated a significant difference in the domain “Perception of management” between professionals (*p* = 0.02), with an increase in the mean score among anesthesiologists after the intervention. In the domain “Perception of professional performance”, anesthesiologists had lower mean scores than nurses (*p*= 0.007), which suggests that nurses’ professional performance is more affected by tiredness and work overload (Table 1).

The LMM displayed a significant effect of the implementation of the PSC/NAP by nurses on the total SAQ/OR score (Table 2), with an average increase of 4.12 points, showing a positive effect on the safety climate after the intervention.

**Table 2.** Effect of the implementation of the PSC/NAP checklist on the Safety Attitudes Questionnaire/Operating Room Version (SAQ/OR) questionnaire after intervention, estimated by the linear effects mixed regression model – São Paulo, SP, Brazil, 2018.

SAQ total post-intervention	Period	β	95% CI*	p-value	
				Main	Interaction
		4.12	–1.44 − 9.68	0.04	
Items executed of PSC/NAP	Pre	0.007	–0.005 − 0.02	0.10	0.02
	Post	–0.007	–0.02 − 0.005		
Age of professional	Pre	0.52	0.17 − 0.87	<0.001	<0.001
	Post	0.26	–0.12 − 0.63		
Position: nurse	Pre	–4.27	–20.74 − 12.19	0.47	0.03
	Post	–2.11	–18.57 − 14.34		
Professional experience	Pre	–0.27	–0.62 − 0.07	0.03	<0.001
	Post	0.22	–0.15 − 0.59		
Sex: Male	Pre	–12.65	–27.82 − 2.52	0.02	<0.001
	Post	–3.62	–18.80 − 11.55		
Number of completed PSC/NAP	Pre	–0.16	–0.37 − 0.04	0.03	<0.001
	Post	–0.11	–0.32 − 0.09		
Anesthesiologist exposure to the PSC/NAP	Pre	0.36	–0.71 − 1.44	0.35	<0.001
	Post	–0.26	−1.33 − 0.82		

* CI = confidence interval; pre: pre-intervention; post: post-intervention.

Different effects of the implementation of the PSC/NAP on the total SAQ/OR questionnaire score in the factors of interest analysed were observed, in particular the reduction of the difference between different ages and improved score among the younger participants after the intervention (Table 2).

The analysis of the position revealed that, among nurses, the regression coefficient ranged from –4.27 to –2.11, indicating an increase in the SAQ/OR score and consequent positive effect among these professionals. For the factors professional experience and males, there were positive variations of the coefficients between the pre- and post-intervention, with a lowering of the difference between the sexes, and better perception among the most experienced professionals. The number of instruments applied presented a post-intervention regression coefficient of –0.11, indicating that the use of the PSC/NAP during anesthesia contributed to the increase of the SAQ/OR score (Table 2).

For each item of the PSC/NAP filled, the SAQ/OR score decreased to –0.007 from the pre- to the post-intervention period among professionals, and exposure of anesthesiologists to the PSC/NAP decreases the SAQ/OR score by –0.26 (Table 2).

In the evaluation of the total score of the TCI instrument, a significant increase in the score for both professional categories was observed in the post-intervention period (*p*= 0.01) (Table 3). The mean score variation of the domains revealed that, among nurses and anesthesiologists, there is evidence of a significant mean change in the pre- and post-intervention periods, in the domain “Participation in the team” domain (*p*= 0.004), and in the domain “Task orientation” (*p*= 0.04).

**Table 3.** Comparison of preoperative and postoperative score of the workers’ perceptions, by position, according to the domains of the instrument “Team Climate Inventory (TCI)” – São Paulo, SP, Brazil, 2018.

Domains	Position	Period		p-values		
		Pre mean (SD*)	Post mean (SD)	Exposition period	Position	Interaction: period and position
Total TCI score	N	150 (28.1)	160 (22.2)	0.01	0.08	0.93
	A	162 (25.8)	172 (25.5)			
Participation in the team	N	38.3 (6.97)	41.8 (6.9)	0.004	0.06	0.75
	A	42.4 (6.77)	45.5 (7.11)			
Support for new ideas	N	24.9 (5.85)	26.8 (4.81)	0.14	0.07	0.75
	A	28.1 (5.12)	29.6 (5.57)			
Team goals	N	55.2 (11.8)	56.1 (8.04)	0.19	0.27	0.63
	A	56.6 (9.52)	59.1 (9.97)			
Task orientation	N	31.6 (8.94)	36.1 (7.15)	0.04	0.11	0.51
	A	34.9 (6.33)	38 (6.77)			

* SD = standard deviation; N: nurse; A: anesthesiologist.

The LMM showed a significant effect of the implementation of the PSC/NAP on the total TCI score, with a mean increase of approximately 60.09 points. The change in score was observed mainly in relation to the factor professional experience, indicating an improvement in the score among the most experienced workers (regression coefficient variation from –0.48 to –0.12, *p*= 0.04) (Table 4).

**Table 4:** Effect of the implementation of the PSC/NAP checklist on the Team Climate Inventory (TCI) and on the domains “Participation in the team” and “Task orientation” of the Team Climate Inventory (TCI) after intervention, estimated by a Linear Mixed-Effects Regression Model – São Paulo, SP, Brazil, 2018.

	Period	p-value		Main	Interaction
		β	95% CI*		
**TCI total post-intervention**		60.09	42.40–77.79	<0.001	
Items executed of PSC/NAP	Pre	0.02	–0.01 − 0.06	0.09	0.02
	Post	–0.02	–0.06 − 0.01		
Age of professional	Pre	–1.26	–2.38– −0.14	0.002	<0.001
	Post	–2.28	–3.46 − −1.09		
Position: nurse	Pre	–31.74	–79.17 − 15.69	0.07	<0.008
	Post	–40.35	–87.73 − 7.03		
Professional experience	Pre	–0.48	–1.58 − 0.62	0.22	<0.04
	Post	–0.12	–1.29 − 1.04		
Sex: Male	Pre	–6.54	–50.20 − 37.12	0.68	0.94
	Post	–6.30	–49.98 − 37.38		
Number of completed PSC/NAP	Pre	–0.17	–0.76 − 0.42	0.41	0.31
	Post	–0.17	–0.75 − 0.42		
Anesthesiologist exposure to the PSC/NAP	Pre	0.05	–3.04 − 3.13	0.41	0.001
	Post	–0.48	–3.57− 2.60			
**Domain: participation in the team**				
**Post-intervention**		27.75	23.20 − 32.30	<0.001	
Items executed of PSC/NAP	Pre	0.003	–0.006 − 0.01	0.36	0.18
	Post	–0.003	–0.01 − 0.006		
Workers’ age	Pre	0.19	–0.09− 0.47	0.06	<0.001
	Post	–0.47	–0.77 – −0.18		
Position: nurse	Pre	–5.26	–14.58 − 4.06	0.12	<0.001
	Post	–9.99	–19.29 − −0.68		
Professional experience	Pre	–0.32	–0.60 − −0.05	0.001	<0.001
	Post	0.18	–0.11 − 0.46		
Sex: Male	Pre	–3.94	–12.50 − 4.62	0.20	0.05
	Post	–2.43	–11.00 − 6.14		
Number of completed PSC/NAP	Pre	–0.05	–0.16 − 0.07	0.26	0.04
	Post	–0.04	–0.16 − 0.07		
Anesthesiologist exposure to the PSC/NAP	Pre	0.20	–0.41 − 0.80	0.36	<0.001
	Post	–0.25	–0.85 − 0.35		
**Domain: Task orientation**				
**Post-intervention**		7.42	3.17 − 11.68	<0.001	
Items executed of PSC/NAP	Pre	0.002	–0.007 − 0.01	0.60	0.46
	Post	–0.002	–0.01 − 0.007		
Workers’ age	Pre	–0.29	–0.56 − −0.02	0.003	<0.001
	Post	–0.55	–0.83 − −0.26		
Position: nurse	Pre	–9.23	–20.54 − 2.07	0.03	<0.001
	Post	–5.38	–16.68 − 5.91		
Professional experience	Pre	–0.03	–0.29 − 0.24	0.79	<0.001
	Post	0.21	–0.07 − 0.49		
Sex: Male	Pre	–3.26	–13.66 − 7.15	0.38	<0.001
	Post	0.20	–10.21 − 10.61		
Number of completed PSC/NAP	Pre	–0.09	–0.23 − 0.05	0.08	<0.001
	Post	–0.05	–0.19 − 0.10		
Anesthesiologist exposure to the PSC/NAP	Pre	–0.12	–0.85 − 0.62	0.65	0.60
	Post	–0.10	–0.83 – 0.64		

* CI = confidence interval; pre: pre-intervention; post: post-intervention.

In the analysis of the domain “Participation in the team”, the factors that contributed to the increase of the score were professional experience, male sex, and number of PSC/NAP completed, indicating a reduction of the score difference between the sexes and improvement among more experienced workers. In the domain “Task orientation”, we can observe the increase of the score between nurses and experienced workers which highlights the improvement in the perception of the performance of individual actions among the team (Table 4).

In the domain “Support for new ideas”, the interaction with the position nurse presented a significant effect (*p*= 0.04) after the intervention, with regression coefficient of –6.33 to –4.67, along with the factor exposure of the anesthesiologist in the implementation of the PSC/NAP with *p* < 0.001 and regression coefficient of –0.46 to –0.10. This indicates that the PSC/NAP contributed positively to the improvement of perception about the introduction of new ideas in the team, but with a greater emphasis on nurses.

The domain “Team goals”, revealed a significant reduction (*p* < 0.001) in the regression coefficients mainly in the nurse position (β = –11.59 to –20.45) and exposure of the anesthesiologist in the implementation of the PSC/NAP(β = 0.22 to –0.19).

## DISCUSSION

The implementation of the PSC/NAP led to changes in the scores about the perception of safety and teamwork climate of nurses and anesthesiologists participating in this study. In countries where there is a lack of national guidelines regulating nursing practice during the anesthetic procedure, the implementation of the measures, such as PSC/NAP, could be the first line to show the positive impact of anesthesia nursing care on patient safety and teamwork and help with the advances of the specialty on nursing anesthesia.

Healthcare culture and safety climate affect organizational performance and can seriously affect patient care and staff. Therefore, cultural changes in the workplace may facilitate evidence implementation^(20)^.

The climate of safety is variable among healthcare institutions, with a score during the implementation of the SAQ-short form version in other realities ranging from a mean of 53.5 in the operating room^(24)^ to means of 61.5 to 69 in different units of the hospital^(25)^.

In relation to the domain “Perception of management”, a difference of perception among professionals (*p* = 0.02) was observed, with the highest score among anesthesiologists after the intervention. Another study showed that the perception of management was one of the more sensitive domains evaluated both for nurses, and doctors, indicating differences among these professionals regarding the actions of institutional management concerning safety^(26)^. Thus, the values and principles of an organisation need to be clearly communicated to the staff, which allows individual employees to compare their values and principles to the organisation’s^(20)^.

Besides, medical professionals receive more support from health services management or hold leadership positions in organizations, which favours a closer evaluation of the measures carried out by the institution regarding quality and safety, and a different perception from other workers^(26)^. On the other hand, nurses not always have access to adverse events notifications in the hospitals and do not participate in the construction of strategies and actions implemented by managers and coordinators to improve the safety processes.

In Brazil, nurses are subjected to the conducts and policies of health services, characterized by the organizational culture, which directly influences the professionals’ work, and possibilities to act in the institutional management. In contrast, Brazilian medical professionals often have no employment relationship with the health service, being mainly service providers. Thus, it is essential to change the traditional paradigms, transforming the hierarchical administration models. Nurses’ work is fragmented and disconnected from managing and caring, for them to have a more dynamic performance in institutional management and decision-making, and collaborate with other health professionals^(27)^.

Differences in scores related to safety were observed between sexes in the surgical environment and among professionals with greater experience, related to less satisfaction with the work carried out^(28)^ and the ability to identify the individual and collective competencies for the commitment and performance of health institutions to patient safety^(25)^. Thus, the reduction of differences in scores related to safety among nurses and anesthesiologists indicated a rise in the SAQ/OR instrument score after the intervention. Although not reaching a score of 75 points, this result shows a positive increase after the implementation of the PSC/NAP.

The implementation of guidelines of care or the highest number of items completed from the surgical safety checklist during the surgical procedure resulted in the improvement of communication among professionals, in the sharing of information, leading to the development of collaborative work, establishing the commitment of its actions in accordance with the measures proposed, and the possibility of communication within the working group to discuss the quality of care provided^(13)^.

Adverse events in anesthesia were related, among other factors, to failures in planning the necessary care for the anesthetic procedure and monitoring the patient, complex communication, and failure to check the equipment^(3)^. The use of a checklist in anesthesia to confer airway materials and test equipment, before anaesthetic induction, collaborated to improve the performance of the professionals who applied the checklist and to increase the exchange of information, with consequent prevention of adverse events^(3)^.

About teamwork, the analysis of the mean variation of the total score of the TCI instrument indicated an increase in the score between the two professional categories after the intervention, with changes, mainly in the domains “Participation in the team” and “Task orientation”, underlining that the duration of professional experience was the main factor associated with the increased score in the TCI instrument.

The interprofessional collaboration process is triggered by patients’ needs and includes integration, trust, respect, openness to collaboration, a feeling of belonging, humility, time to listen and talk. Interprofessional collaboration requires communication and shared workspaces to ensure frequent contact and sociability, appreciation and knowledge of different practices and professional roles, and shared leadership to deal with conflicts and tensions^(29)^.

The domain “Team goals” revealed a significant reduction (*p* < 0.001) in the regression coefficients, because although nurses recognised the potential of the PSC/NAP to guide their activities, the actions may not necessarily establish common goals within the team, leading to the perception of gaps between the goals to be achieved and the care established by the anesthesiologist during the anaesthetic procedure and how the nursing team can influence this process^(30)^. Also, although the goals of research were explained to all anesthesiologists included in this study, some professionals may disagree that there is an important role of nurses during anesthesia.

Team’s adaptive capacity may be under additional stress factors when different team members alternate during their shifts, as lack of knowledge about one another may increase miscommunications and interruptions during surgical procedures. Nurses wish to work collaboratively with physicians to coordinate patient care, in a scenario where there is a clear opening for communication among professionals, allowing equity in the decision-making process and suggestions for implementing the care plan. Good communication patterns are experienced when each professional perceives to be involved in a shared challenge and when an individual’s expertise is valued by each member^(30)^.

Positive changes were observed in relation to the use of the PSC/NAP to improve the perception of safety and teamwork, indicate the importance of the use of the tool by nurses in the operating room to improve daily practices of Brazilian nurses, as well as the need for long-term monitoring of the benefits of its use in the care of surgical patients.

Future research is required about the role of nurses in anesthesia in Brazil, and the impact of standardized nursing anesthesia actions, the use of the PSC/NAP to reduce adverse events in anesthesia and strengthen collaborative work between nurses and anesthesiologists in the country.

### Limitations

The lack of randomization and control group, and the data collection executed in one institution, evaluating the perception of a single team, could be limitations of this study. Furthermore, the length of data collection could be a limitation, because 80–100% adherence to new standards of practice is expected after one year of implementation^(20)^.

## CONCLUSION

The study showed a change in the perception score of safety and teamwork climate among nurses and anesthesiologists assessed after the intervention, indicating that the PSC/NAP may foment collaborative work among professionals and contribute to safety practices during anesthesia procedures.

## References

[1] Braz LG, Braz JRC, Modolo MP, Corrente JE, Sanchez R, Pacchioni M (2020). Perioperative and anesthesia-related cardiac arrest and mortality
rates in Brazil: a systematic review and proportion
metaanalysis. PLoS One.

[2] Braghiroli KS, Braz JRC, Rocha B, El Dib R, Corrente JE, Braz MG (2017). Perioperative and anesthesia related cardiac arrests in geriatric
patients: a systematic review using meta-regression analysis. Sci Rep.

[3] Lemos CS, Poveda VB (2019). Adverse events in anesthesia: an integrative
review. J Perianesth Nurs.

[4] Curatolo CJ, McCormick PJ, Hyman JB, Beilin Y (2018). Preventable anesthesia-related adverse events at a large tertiary
care center: a nine-year retrospective analysis. Jt Comm J Qual Patient Saf.

[5] Liberman JS, Slagle JM, Whitney G, Shotwell MS, Lorinc A, Porterfield E (2020). Incidence and classification of nonroutine events during
anesthesia care. Anesthesiology.

[6] Alsalem G, Bowie P, Morrison J (2018). Assessing safety climate in acute hospital settings: a systematic
review of the adequacy of the psychometric properties of survey measurement
tools. BMC Health Serv Res.

[7] Tseng HM, Liu FC, West MA (2009). The Team Climate Inventory (TCI): a psychometric test on a
taiwanese sample of work groups. Small Group Res.

[8] Khan FA, Merry AF (2018). Improving anesthesia safety in low-resource
settings. Anesth Analg.

[9] Haugen AS, Sevdalis N, Softeland E (2019). Haugen AS, Sevdalis N, Softeland E. Impact of the World Health
Organization surgical safety checklist on patient safety. Anesthesiology.

[10] Yu D, Zhao Q (2020). Effects of a perioperative safety checklist on postoperative
complications following surgery for gastric cancer: a single-center
preliminary study. Surg Innov.

[11] Haugen AS, Søfteland E, Sevdalis N, Eide GE, Nortvedt MW, Vincent C (2020). Impact of the Norwegian National Patient Safety Program on
implementation of the WHO Surgical Safety Checklist and on perioperative
safety culture. BMJ Open Qual.

[12] Kilbane H, Oxtoby C, Tivers MS (2020). Staff attitudes to and compliance with the use of a surgical
safety checklist. J Small Anim Pract.

[13] Willassen ET, Jacobsen ILS, Tveiten S (2018). Safe surgery checklist, patient safety, teamwork, and
responsibility-coequal demands? a focus group study. Glob Qual Nurs Res.

[14] Lourenção DCA, Tronchin DMR (2018). Safety climate in the surgical center: validation of a
questionnaire for the Brasilian scenario. Rev Eletr Enf.

[15] Haugen AS, Waehle HV, Almeland SK, Harthug S, Sevdalis N, Eide GE (2019). Causal analysis of World Health Organization’s surgical safety
checklist implementation quality and impact on care processes and patient
outcomes: secondary analysis from a large stepped widge cluster randomized
controlled trial in Norway. Ann Surg.

[16] Saxena S, Krombach JW, Nahrwold DA, Pirracchio R (2020). Anaesthesia-specific checklists: a systematic review of
impact. Anaesth Crit Care Pain Med.

[17] Lemos CS, Peniche ACG (2016). Nursing care in the anesthetic procedure: an integrative
review. Rev Esc Enferm USP.

[18] Lemos CS, Poveda VB (2022). Role of perioperative nursing in anesthesia: a national
overview. Rev Esc Enferm USP.

[19] Lemos CS, Poveda VB, Peniche ACG (2017). Construction and validation of a nursing care protocol in
anesthesia. Rev Lat Am Enfermagem.

[20] Munn Z, McArthur A, Porritt K, Lizarondo L, Moola S, Lockwood C, Porritt K, McArthur A, Lockwood C, Munn Z (2020). JBI Handbook for evidence implementation.

[21] Lemos CS, Poveda VB (2021). Situação problema: metodologia ativa para ação educativa sobre
anestesia com enfermeiros de centro cirúrgico. Revista SOBECC.

[22] Pinheiro JPA, Uva AS (2016). Safety climate in the operating room: translation, validation and
application of the safety attitudes questionnaire. Port J Public Health.

[23] Silva MC, Peduzzi M, Sangaleti CT, Silva D, Agreli HF, West MA (2016). Cross-cultural adaptation and validation of the teamwork climate
scale. Rev Saude Publica.

[24] Carvalho PA, Gottems LBD, Pires MRGM, Oliveira ML (2015). Safety culture in the operating room of a public hospital in the
perception of healthcare professionals. Rev Lat Am Enfermagem.

[25] Carvalho REFL, Arruda LP, Nascimento NKP, Sampaio RL, Cavalcante ML, Costa AC (2017). Assessment of the culture of safety in public hospitals in
Brazil. Rev Lat Am Enfermagem.

[26] Göras C, Unbeck M, Nilsson U, Ehrenberg A. (2017). Interprofessional team assessments of the patient safety climate
in Swedish operating rooms: a cross-sectional survey.. BMJ Open..

[27] Schirle L, Norful AA, Rudner N, Poghosyan L. (2020). Organizational facilitators and barriers to optimal APRN
practice: an integrative review.. Health Care Manage Rev..

[28] Yang YM, Zhou LJ. (2021). Workplace bullying among operating room nurses in China: A
cross-sectional survey.. Perspect Psychiatr Care..

[29] Sangaleti C, Schveitzer MC, Peduzzi M, Zoboli ELCP, Soares CB. (2017). Experiences and shared meaning of teamwork and interprofessional
collaboration among health care professionals in primary health care
settings: a systematic review.. JBI Database System Rev Implement Rep..

[30] Misseri G, Cortegiani A, Gregoretti C. (2020). How to communicate between surgeon and
intensivist?. Curr Opin Anaesthesiol..

